# Asymmetric Isoporous Membranes of 2-Vinylpyridine-Styrene Linear Diblock Copolymers: Fabrication and Evaluation in Water Treatment

**DOI:** 10.3390/polym18020149

**Published:** 2026-01-06

**Authors:** Maria Rikkou-Kalourkoti, Katerina Antoniou, Nicholas A. Pissarides, Georgios T. Papageorgiou, Costas S. Patrickios

**Affiliations:** 1 Department of Chemistry, University of Cyprus, P.O. Box 20537, Nicosia 1678, Cyprus; hsc.rm@frederick.ac.cy; 2 State General Laboratory, 44 Kimonos Street, Nicosia 1451, Cyprus; kantoniou@sgl.moh.gov.cy (K.A.); npissarides@sgl.moh.gov.cy (N.A.P.); georgiospapageorgiou@cytanet.com.cy (G.T.P.)

**Keywords:** reversible addition-fragmentation chain transfer polymerization, amphiphilic diblock copolymers, self-assembly nanostructures, asymmetric isoporous membranes, water treatment, ultrafiltration

## Abstract

Herein, we report the synthesis via controlled reversible addition-fragmentation chain transfer (RAFT) polymerization of amphiphilic 2-vinylpyridine-*b*-styrene (2VPy-*b*-Sty) diblock copolymers of high molar masses (range: 52,100–304,000 g mol^−1^) and various compositions (range: 2VP content 11.6–59.2 mol%) and their use for the fabrication of nanoporous membranes. The successful synthesis of the amphiphilic diblock copolymers was confirmed through the characterization of their molar masses, molar mass distribution, and composition using GPC and ^1^H-NMR spectroscopy, respectively. Subsequently, membranes of the diblock copolymers were fabricated following the “phase inversion” technique. The resulting membranes were characterized via scanning electron microscopy which revealed the presence of sphere percolation networks morphology for all diblock copolymers with *M_n_* ranging from 120 to 300 kDa and 2VPy content between 10 and 15 mol% at the optimal conditions. Afterward, the developed membranes were evaluated in terms of their permeability towards water and in terms of their ability to retain two different microorganisms, namely, *Enterococcus faecalis* and *Escherichia coli*, that are known to be harmful to human health. The experimental water flux for a membrane with pore size around 60 nm was equal to 31,400 L h^−1^ m^2^ and expectedly decreased with the decrease in membrane pore diameter. The retention ability of membranes for *Enterococcus faecalis* and *Escherichia coli* was higher than 90%. In particular, the retention ability for *Enterococcus faecalis* was equal to 98.9% and for *Escherichia coli* was 91.4%. The toxicity of the produced membrane was also determined, and the measured value was relatively low, at 17%.

## 1. Introduction

Despite covering the majority of the planet’s surface, the accessible freshwater supply accounts for under 1% of Earth’s total water resources. In fact, in many countries, people do not have access to clean drinking water. This scarcity of water is the reason why water-related diseases are one of the leading causes of deaths for children under the age of five [1]. Thus, water treatment is no longer a luxury, but a necessity. One physical process for treating water is ultrafiltration, which is used for the removal of bacteria and macromolecules. The pore size in ultrafiltration membranes typically ranges from 5 to 500 nm. These membranes can be produced via track etching, phase inversion, or stretching [2]. Of the three above mentioned techniques, phase inversion has a key advantage over the other two, which is the rapid, one-step, low-cost formation of a stable and selective membrane layer with high porosity. This technique utilizes amphiphilic block copolymers and exploits their self-assembly in selective solvents [3,4,5].

Due to their dual solubility characteristics, amphiphilic block copolymers tend to undergo spontaneous organization into distinct nanoscale structures [6,7,8,9]. This self-assembly behavior is affected by many factors, but the architecture and the composition of the copolymers are the most important ones [10]. Another important factor is the molecular weight (MW), with high MWs facilitating this self-assembly and making the microphase boundaries sharper [11]. This amphiphilic co-polymer self-assembly can be exploited for the fabrication of porous polymeric membranes. These membranes can be symmetrical, consisting of only one porous layer, or asymmetric, consisting of two layers (each having its own porosity) called isoporous membranes [2]. The phase inversion method leads to the formation of asymmetric isoporous membranes with a thin selective top layer connected to a spongy bottom support layer.

Poly(Styrene)-poly(vinyl pyridine) diblock copolymers stand out for their ease of microphase separation, arising from the high incompatibility between the two types of monomer repeating units [4]. For this reason, this type of diblock copolymers represent a system frequently studied for the preparation of polymeric membranes. In most examples reported in the literature, the 4-vinyl pyridine isomer is employed [4,11,12,13,14,15,16], with only a limited number of examples utilizing the 2-vinyl pyridine isomer in its diblock copolymers with styrene [17,18]. For all examples, anionic polymerization was used for the preparation of these diblock copolymers. However, the strict experimental conditions of this polymerization technique—arising from its sensitivity to moisture, oxygen, and carbon dioxide and the requirement for conduction at low temperature—limit its appeal. In contrast, living radical polymerization can be performed under more forgiving conditions, including a wider range of solvents and temperatures, and is often more versatile and applicable to a broader range of monomers.

Among controlled radical polymerization techniques, reversible addition-fragmentation chain transfer (RAFT) polymerization is particularly advantageous for the synthesis of pyridine-containing block copolymers. Vinylpyridine monomers are strongly polar and possess coordinating nitrogen atoms, which can interfere with metal-based controlled radical polymerization methods such as atom transfer radical polymerization (ATRP) and electrochemically mediated ATRP (eATRP) through catalyst coordination or deactivation. In contrast, RAFT polymerization is a metal-free process and exhibits excellent tolerance toward polar and coordinating monomers, allowing high monomer conversions and reliable control even at very high degrees of polymerization. These features render RAFT especially suitable for the preparation of high-molecular-weight pyridine-based block copolymers with well-defined compositions and architectures [19,20].

In the literature, only one example exists of synthesizing diblock copolymers utilizing styrene and 3-vinylpyridine isomers via surfactant-free water-based reversible addition-fragmentation chain transfer (RAFT) emulsion polymerization. This process involves polymerization-induced self-assembly (PISA), resulting in the formation of precisely defined micellar nanoscale structures comprising a polystyrene (PS) core and a poly(3-vinylpyridine) (P3VP) corona facilitated by the macroRAFT agent. This method showcases controlled RAFT polymerization, enabling the fabrication of diblock copolymers with high monomer conversion and showcasing microphase-separated structures dictated by their composition [21].

Thus, the aim in this investigation is to bridge this gap through the synthesis of high-MW linear amphiphilic 2VPy-Sty diblock copolymers of various compositions via controlled RAFT polymerization, which are utilized for the fabrication of nanoporous membranes using the “phase inversion” technique; we also evaluate fabricated membranes in terms of their permeability and their ability to retain harmful microorganisms.

The diblock copolymers synthesized herein represent the first example of such diblocks where the 2VPy block is polymerized first, and they have the highest MWs reported until now for Sty-VPy block copolymers synthesized by RAFT polymerization, reaching up to 300,000 g mol^−1^ [22,23,24,25]. These diblock copolymers were successfully employed for the fabrication of asymmetric isoporous membranes using the “phase inversion” technique with sphere percolation network morphology and a pore diameter of around 50 nm. Evaluation of the produced membranes in water treatment revealed low toxicity and high retention ability for *Enterococcus faecalis* and *E. coli*, being higher than 90%.

## 2. Experimental Procedure

**Materials:** The monomers styrene (Sty, 99%) and 2-vinylpyridine (2VPy, 97%), calcium hydride (CaH_2_, 95%), 2,2-diphenyl-1-picrylhydrazyl hydrate (DPPH, 95%), and toluene (99%) were purchased from Aldrich, Taufkirchen, Germany. Further, 2,2′-Azobis(isobutyronitrile) (AIBN, 95%), *N*,*N*-dimethylformamide (DMF, 96%) and deuterated chloroform (CDCl_3_) were purchased from Merck, Darmstadt Germany. Tetrahydrofuran (THF, 99.8%, both HPLC- and reagent-grade) was purchased from Scharlau, Barcelona Spain. Finally, 2-cyanoprop-2-yl dithiobenzoate (CPDB) was synthesized according to the published procedure [26] and was used as the chain transfer agent (CTA) for the RAFT polymerizations.

**Methods:** The monomers Sty and 2VPy were initially purified by passing them through columns packed with basic alumina to eliminate any acidic contaminants and residual inhibitors of free radical polymerization. Following this, DPPH was introduced as a stabilizer to suppress unwanted thermal polymerization. To remove remaining moisture, calcium hydride was added, and the mixture was stirred overnight. The monomers were then subjected to vacuum distillation to eliminate both DPPH and any residual impurities. AIBN, used as the radical initiator, underwent two recrystallizations from ethanol to ensure its purity. The solvent DMF was dried over CaH_2_ and distilled under a vacuum immediately before use to maintain anhydrous conditions.

**RAFT Polymerizations:** Synthesis of Linear Diblock Copolymers. Linear diblock copolymers of 2VPy-Sty were prepared using stepwise RAFT polymerization: the 2VPy block was first synthesized and then extended with Sty. As an example, we detail below the polymerization procedure followed for the preparation of the 2VPy_222_-*b*-Sty_1092_ diblock copolymer. To a 25 mL dry Schlenk flask, 5 mL 2VPy (4.89 g, 0.046 mol), 34.3 mg CPDB (1.54 × 10^−4^ mol), 10.1 mg AIBN (6.19 × 10^−5^ mol), and 1.6 mL of DMF were added. After complete dissolution of the reagents, three freeze–evacuate–thaw cycles were performed to the mixture, and the Schlenk flask was placed in an oil bath at 65 °C for 20 h. After purification of the linear homopolymer (2VP monomer conversion to polymer = 74%) via two precipitations in hexane and drying for three days in a vacuum oven, it was used as macroCTA for the preparation of the diblock copolymer. To this end, to a 25 mL dry Schlenk flask, 24.4 mL Sty (22.08 g, 0.21 mol), 3 g of CPDB-2VPy_222_ macroCTA (7.85 × 10^−5^ mol, polymer number-average MW = *M_n_* = 38,200 g mol^−1^), and 8.0 mg AIBN (4.90 × 10^−5^ mol) were added. After complete dissolution of the reagents, three freeze–evacuate–thaw cycles were performed to the mixture, and the Schlenk flask was placed in an oil bath at 65 °C for 22 h. The resulting 2VPy_222_-*b*-Sty_1692_ diblock copolymer (styrene monomer conversion to polymer = 72.9%) was isolated after two precipitations in methanol.

**Membrane Fabrication:** Asymmetric isoporous membranes of the 2VPy-Sty diblock copolymers were fabricated via the “phase inversion” technique, involving three steps. In the first step, polymer solutions in THF, DMF and dioxane mixtures with concentrations ranging from 13 to 20% wt. were cast onto a glass plate using a doctor’s blade. In the second step, the formed film was kept for a few seconds (5 to 20 s) under air. In the third and final step, the glass plate with the formed film was immediately immersed in a water bath. The immersion of the film in the water bath resulted in solvent–non-solvent exchange and the formation of the membrane pores.

**Polymer Characterization:** The molecular weight distributions (MWDs) of all synthesized polymers were determined via gel permeation chromatography (GPC) utilizing a refractive index (RI) detector (hereafter referred to as GPC-RI). Analyses were performed using a single PL-Mixed D column (Polymer Laboratories), with tetrahydrofuran (THF) as the eluent delivered at a constant flow rate of 1.0 mL/min by a Waters 515 isocratic pump. Detection of eluted species was carried out using a Polymer Laboratories ERC-7515A RI detector. Proton NMR spectra were recorded in CDCl_3_ on a 500 MHz Bruker Avance spectrometer fitted with an Ultrashield magnet, enabling structural confirmation of both homopolymers and copolymers.

**Micelle Characterization**: *Atomic Force Microscopy (AFM)*. Topographic imaging of the polymeric micelles was performed using a PicoPlus scanning probe microscope (Molecular Imaging, Agilent, Santa Clara, CA, USA) equipped with a cantilever supplied by Applied Nanostructures. The cantilever featured a length of 125 μm, width of 45 μm, resonant frequency between 200 and 400 kHz, spring constant ranging from 25 to 75 N·m^−1^, tip radius under 10 nm, and a height of 12–16 μm. Dilute solutions (0.001% wt) of the amphiphilic diblock copolymers 2VPy–Sty were prepared in chloroform or toluene and applied to a highly ordered pyrolytic graphite (HOPG) substrate by spin-coating at 102 rps for one minute. Imaging was performed in tapping mode under ambient conditions at room temperature. All AFM images were acquired using identical scanning conditions; using a constant scan rate in the range typically employed for tapping-mode imaging, and the cantilever specifications are provided above.

*Dynamic Light Scattering (DLS).* Hydrodynamic diameters of the micelles formed in either toluene or chloroform (1% *w*/*w*) were determined using a 90Plus Brookhaven DLS instrument, Nashua, NH, USA, equipped with a BI-9000 correlator and a 30 mW red diode laser (λ = 673 nm). Scattering data were collected at a fixed angle of 90° under ambient conditions. To prepare the solutions, 60 mg of each diblock copolymer was dissolved in 6.0 g of the selected solvent. Before analysis, solutions were passed through 0.45 μm PTFE filters and allowed to stand for a minimum of one hour to eliminate residual air bubbles. Each sample was analyzed over five runs of two minutes each, and the resulting data were averaged. Size distributions were extracted using multimodal fitting based on non-negatively constrained least squares (NNCLS) analysis.

Theoretical estimations of micellar size limits were calculated to support the experimental DLS and AFM data. The maximum possible micellar diameter was estimated assuming fully extended polymer chains arranged in a spherical morphology by multiplying the total degree of polymerization (DP) by 0.252 nm (the length of a single repeating unit) and converting the resulting radius to diameter. The lower size limit corresponding to single-chain (unimer) dimensions was estimated using random flight statistics, accounting for the tetrahedral carbon bond angle and incorporating a stiffness factor of 2.44 for polystyrene. The root mean square diameter of gyration was calculated according to ⟨dg^2^〉^1/2^ = 2 × (2.44 × 2 × DP/3)^1/2^ × 0.154, where 0.154 nm corresponds to the C–C bond length. These calculations provide theoretical size boundaries for comparison with experimentally determined micellar diameters.

**Membrane Characterization**: *Scanning Electron Microscopy (SEM).* The morphology of the polymeric membranes was characterized using a Vega TS5136LS-Tescan scanning electron microscope at a voltage of 20 kV. Prior to the SEM imaging, the samples were gold-sputtered (~15 nm thickness) using the K575X Turbo sputter coater–Emitech sputtering system, Quorum Technologies Ltd., West-Sussex, UK.

*Toxicity of the Membranes.* The toxicity of the polymeric membranes was determined by spreading 0.1 mL of solution containing *Enterococcus faecalis* at a concentration of 100 colony-forming units/mL on the membrane. The membrane toxicity toward *Enterococcus faecalis* was calculated from Equation (1). The procedure was repeated three times.

(1) $$Toxicity=1-\frac{\#colonies\;grown\;on\;membrane}{\#colonies\;placed\;on\;membrane}$$

*Retention Ability of the Membranes*. The performance of the membranes in retaining microorganisms that are harmful to human health was tested by charging 20 mL water with 0.5 mL of microorganisms. Two microorganisms were tested: *Escherichia coli* and *Enterococcus faecalis*. After filtration of the suspension using the fabricated membranes, the polymeric membranes were placed on a proper medium and incubated at the proper temperature to develop pink colonies. The filtrate was filtered again with a commercially available 0.45 μm millipore membrane that was also placed in a selective medium and incubated. In the case of *Enterococcus faecalis*, the membranes were placed on *Slanetz and Bartley* medium and incubated at 37 °C for 48 h. In the case of *Escherichia coli*, the membranes were placed on L-TTC medium and incubated at 35 °C for 24 h. For both microorganisms, the procedure was repeated five times, and the retention ability was calculated from Equation (2).

(2) $$Retention\;ability=\frac{\#colonies\;on\;membrane}{\#colonies\;on\;membrane+\#colonies\;in\;filtrate}$$

## 3. Results and Discussion

***Optimization of Polymerization Conditions:*** Before proceeding to the synthesis of the desired diblock copolymers, a preliminary study was performed to optimize the controlled RAFT polymerization of the 2VPy monomer using 2-cyanoprop-2-yl dithiobenzoate (CPDB) as a chain transfer agent (CTA). In particular, kinetic studies were performed to check how the relative loadings of AIBN initiator, CPDB CTA and 2VP monomer influenced monomer conversion to polymer and polymer quality, when aiming for large 2VPy homopolymers with a targeted DP of 500.

**Figure 1:**
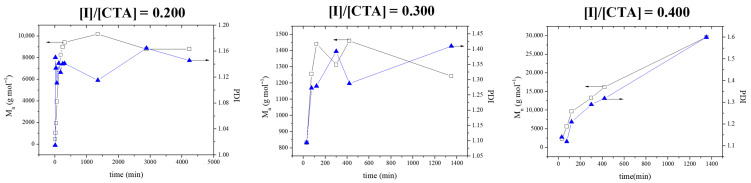
Temporal evolution of the number-average molar mass (*Mn*, **black line**) and dispersity (Đ, **blue line**) for growing poly(2VPy) for three different initiator-to-CTA molar ratios at a constant targeted degree of polymerization (DP = 500).

The first parameter that was examined was the molar ratio of the AIBN initiator to the CPDB CTA. Three different molar ratios of initiator to CTA were used (0.2, 0.3, and 0.4) at a constant targeted 2VPy degree of polymerization equal to 500. Figure 1 presents the time dependence of the *M_n_*s and *Đ*’s in the three cases. The figure shows that the highest *M_n_* values were obtained when the molar ratio of initiator to CTA was equal to 0.400. Despite the high *Đ* values obtained at this molar ratio, this was the only case in which monomer conversion reached 70%; in the other two cases, monomer conversion reached only ~15%. Thus, this highest initiator-to-CTA molar ratio was chosen as optimal for the polymerizations.

The effect of monomer concentration on polymerization was examined next. Three monomer concentrations of 5 M, 7 M and bulk monomer (=9.3 M) were tested. The polymerizations were performed at the 0.4 initiator-to-CTA molar ratio and targeted DP of 500.

**Figure 2:**
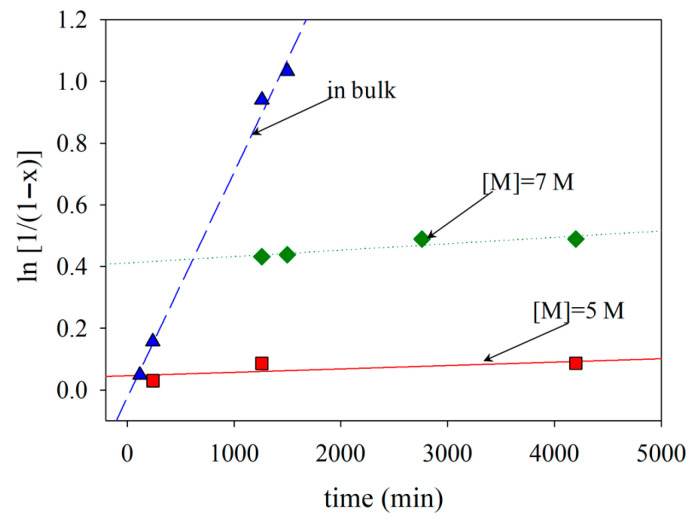
Kinetics of the RAFT polymerization of 2VPy at three different monomer concentrations at 65 °C using [Initiator]/[CTA] = 0.400 and [Monomer]–[Initiator] = 500.

The kinetics of the polymerization for the three monomer concentrations (in bulk, 7 M and 5 M) are shown in Figure 2. The first-order kinetic plots are linear, indicating a constant radical concentration throughout the polymerization. The polymerization times increased with a reduction in monomer concentration. When monomer concentration decreases in raft polymerization, the rate of reaction slows down due to fewer monomer molecules available to react. This decrease in monomer concentration reduces the frequency of encounters between the initiating radicals and monomer molecules, resulting in a slower initiation step. As a result, the overall kinetics of raft polymerization decrease, leading to a slower rate of polymerization. When the lowest 2VPy monomer concentrations were employed, 5 or 7 M polymers with low *Đ* values were obtained. However, at 5 M monomer concentration, monomer conversion was below 40%. Thus, the 7 M monomer concentration was taken as optimal for the polymerizations. As shown in Figure 1, the optimized polymerization conditions lead to unimodal molecular weight distributions with dispersity values around 1.5. While such dispersity values are higher than those typically observed for low-molecular-weight RAFT systems, they are characteristic of RAFT polymerizations targeting very high molecular weights. Importantly, the absence of multimodal distributions indicates uniform chain growth, which is a key prerequisite for homogeneous self-assembly and contributes to the formation of membranes with uniform pore size and morphology during the phase inversion process.

***Synthesis and Characterization of Amphiphilic Diblock Copolymers:*** Amphiphilic diblock copolymers were prepared by extending the homopolymers of 2VPy synthesized at the optimal polymerization conditions, with styrene (Sty) in the bulk, at 120 °C. The family comprised copolymers with different Sty contents. All amphiphililc diblock copolymers were characterized in terms of their *M_n_*s and their compositions using GPC-RI and ^1^H-NMR spectroscopy, respectively. The results of these characterizations are presented in Table 1.

The determined *M_n_* values were slightly lower than the theoretical ones in the cases of copolymers, whereas the reverse was observed in the cases of homopolymers. In all cases, the MWDs were found to be relatively narrow, with *Đ* values around 1.5. The compositions of the copolymers determined from the ^1^H NMR spectra were found to be very close to the theoretically calculated percentages (for the calculation of the theoretical composition, monomer conversion was taken into account), indicating good structural control during RAFT polymerization. For RAFT polymerizations targeting very high molecular weights (up to ~300,000 g mol^−1^), dispersity values around 1.4–1.6 are commonly reported and are consistent with controlled radical polymerization behavior. For this study, Sty-*b*-2VPy amphiphilic copolymers were also prepared. When pSty was used as macro CTA, the experimental values for composition and MW were different from the theoretical ones, thus indicating that better control of the polymerization was achieved when the VPy block was prepared first. When the hydrophilic block (2VPy) is polymerized first, it allows for efficient initiation and propagation of the polymer chain with minimal termination or transfer reactions. This is because the hydrophilic monomer typically has higher reactivity in RAFT polymerization compared to the hydrophobic monomer. As a result, the polymerization proceeds smoothly, leading to better control over the molecular weight and composition of the hydrophilic block. In all previous syntheses of such diblocks, the Sty block was polymerized first, and the highest MW reported for those Sty-VPy diblock copolymers synthesized by RAFT polymerization was around 30,000 g mol^−1^ [18]. Herein, we report the first example of linear synthesis of amphiphilic diblock copolymers based on the 2VPy-Sty block sequence via RAFT polymerization with molecular weights of up to 300,000 g mol^−1^.

***Self-assembly Behavior:*** The amphiphilicity of the diblock copolymers leads to their self-assembly in selective solvents. This self-assembly was investigated via DLS and AFM. In particular, DLS was employed to determine the hydrodynamic diameters of the micelles formed by the present diblock amphiphilic copolymers in toluene and chloroform before and after the addition of methyl iodide. The thus-determined hydrodynamic diameters are listed in Table 2.

The same table presents the estimated size range of micelles formed by the amphiphilic diblock copolymers, assuming fully extended chains arranged in spherical morphology. These limits were calculated by multiplying the total degree of polymerization (DP) of each linear copolymer by 0.252 nm—the length attributed to a single repeating unit [27]—and then doubling the result to convert the maximum radius into the corresponding diameter. In most cases, the samples presented two peak maxima in the hydrodynamic diameter distribution, and the maxima of the most populated peak in each sample appear in bold. In the case of chloroform, the most populated peaks in both the quaternized and the non-quaternized state were very small compared to the maximum possible micellar diameters, suggesting that the copolymers exist as unimers in chloroform with which all monomer repeating units are compatible. When toluene was used, the most populated peaks were much larger than those measured in chloroform and reasonably lower than the maximum possible ones, indicating the formation of well-defined micelles in toluene. An exception was the first diblock copolymer whose 2VPy content was only 11.6%. Quaternization resulted in the shrinkage of the micelles because of the enhanced incompatibility between toluene and the core-forming quaternized 2VPy block. Additionally, the hydrodynamic diameters of the diblock copolymers in toluene increased with the size of the polySty block, as all these copolymers shared the same core forming poly2VPy block.

**Figure 3:**
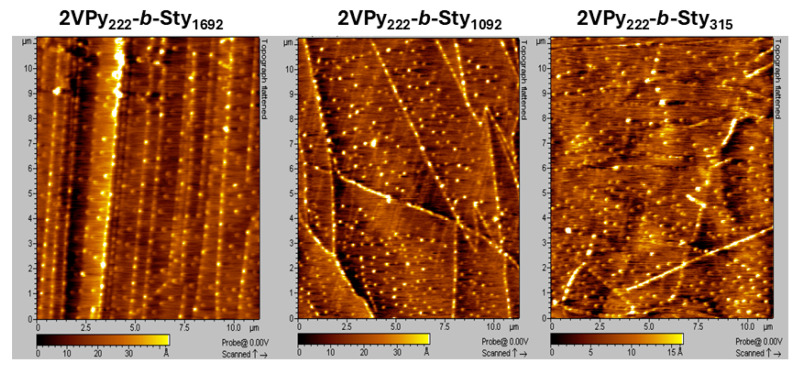
AFM height (topography) images of the amphiphilic linear diblock copolymers 2VPy_222_-*b*-Sty_1692_, 2VPy_222_-*b*-Sty_1092_, and 2VPy_222_-*b*-Sty_315_ deposited from dilute toluene solutions onto HOPG substrates. In all three images, the micelles exhibit uniform size and morphology. The scanned area is indicated by the scale bar shown in each image. All AFM images were acquired in tapping mode under identical scanning conditions.

The diameters of the surface-absorbed micelles formed by the present amphiphilic diblock copolymers were also studied via AFM by depositing diluted copolymer solutions in toluene on a graphite surface. Figure 3 presents the micelles formed by the diblock copolymers. In all three images, the micelles exhibit a uniform size.

The thus-determined adsorption diameters are listed in Table 3. The same table shows the upper limit of the size of the micelles of the amphiphilic copolymers calculated as described before, as well as the lowest limit corresponding to the diblock copolymers’ single chains (unimers). In all cases, the adsorption diameters were larger than the lowest limit and smaller than the maximum possible micelle size. Additionally, the adsorption diameters were larger than the hydrodynamic diameters in toluene due to micellar flattening upon adsorption. Additionally, as shown in Table 3, the adsorption diameters increased with the size of the polySty block, as also observed in DLS.

***Membrane Fabrication and Optimization*:** After their successful synthesis and characterization, the 2VPy-Sty diblock copolymers were used for the fabrication of asymmetric polymeric membranes via the “phase inversion” technique. “Phase inversion” first involved the preparation of a polymer solution which was subsequently cast on a glass plate. The thus-formed film was left for a few seconds exposed to air before being immersed in a water precipitation bath. During the exposure to air, the more volatile solvent, THF, predominantly evaporated, making the solvent mixture richer in DMF and, consequently, more selective for the 2VPy block, leading to the formation of ordered structures. The subsequent immersion of the film in the water precipitation bath resulted in solvent–non-solvent exchange, freezing the ordered top layer. The solvent–non-solvent exchange below this surface was slowed down, leading to the formation of the sponge-like structure under the dense top layer. Importantly, the kinetics of solvent evaporation and subsequent solvent/non-solvent exchange play a decisive role in determining membrane morphology. Rapid solvent–water exchange at the membrane surface kinetically freezes the microphase-separated structure, leading to well-defined pores in the selective layer, whereas slower exchange in the underlying region allows for further polymer rearrangement and results in a sponge-like substructure. Variations in exchange kinetics therefore directly influence pore size, pore density, and overall membrane uniformity. Thus, parameters, such as the solvent mixture composition, the polymer concentration, and the evaporation time, have crucial roles to play in the formation of the asymmetric membranes. Since the copolymer of the present study is new regarding membrane fabrication, those parameters had to be optimized in order to succeed in the formation of well-ordered polymeric membranes. Another important parameter influencing the well-ordering of the membranes is the composition of the diblock copolymers. Thus, only diblock copolymers with 2VPy contents ranging between 10 and 30 mol%, which have the ability to form spheres or cylinders, were used in the optimization studies.

The first parameter that was tested for the preparation of the polymeric membranes was the composition of the solvent mixture into which the block copolymers were dissolved. For Sty-VPy diblock copolymers, binary or ternary solvent mixtures containing DMF and THF are usually used [4,11,12,13,14,15,16,17,18], since THF dissolves both the PS and the PVPy blocks, while DMF is a much better solvent for the polyVPy block. Thus, a two-component solvent mixture composed of DMF and THF at different volume ratios of 70–30, 60–40, and 50–50 were tested first. The polymer concentration and the evaporation time were kept constant at 20% wt and 20 s, respectively. When 30 *v*/*v*% THF was used in the solvent mixture, no membranes were formed. Increasing the THF content to 40 or 50 *v*/*v*% led to the formation of membranes, but these membranes had no pores. THF is the more volatile component in the solvent mixture, and its evaporation is therefore expected to form the channels on the surface of the membrane. Thus, according to the literature, a higher THF volume fraction may favor a more extensive pore formation [17,18]. Another way to control the growth of the channels is the addition of a solvent of intermediate volatility in the solvent mixture, such as dioxane [17]. Thus, when a ternary solvent mixture of equal parts THF, DMF, and dioxane was used, the formation of large spheres was observed. The presence of dioxane (which is a solvent that evaporates faster than DMF but slower than THF) allows the controlled growth of channels. Thus, further optimization with respect to polymer concentration and evaporation time was performed using this ternary solvent mixture.

**Figure 4:**
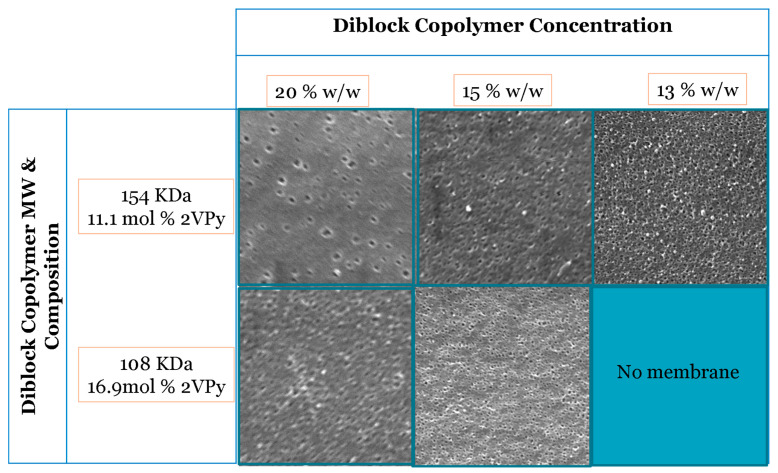
SEM images of the membrane surface morphology fabricated from 2VPy–Sty diblock copolymers using a ternary DMF/THF/dioxane solvent mixture (33:33:33 *v*/*v*) at a constant evaporation time of 20 s and different copolymer concentrations. All SEM images were acquired at a magnification of ×50,000. The images illustrate the effect of polymer concentration on pore formation and surface morphology. Representative pore size values determined from SEM image analysis are discussed in the text and summarized in Table 4.

The second parameter that was examined was the concentration of diblock copolymers in the casting solution at a constant evaporation time of 20 s for the two diblock copolymers selected. The SEM images of these membranes are presented in Figure 4. The results indicated that more pores were formed at lower copolymer concentrations. This can be attributed to the fact that at a higher copolymer concentration (30% *w*/*w*), the mobility of the polymer chains is restricted, making channel formation more difficult. On the other hand, at copolymer concentrations lower than 13 % wt, no membranes could be formed. From the above results, a polymer concentration of around 15% wt. was chosen as optimal.

**Figure 5:**
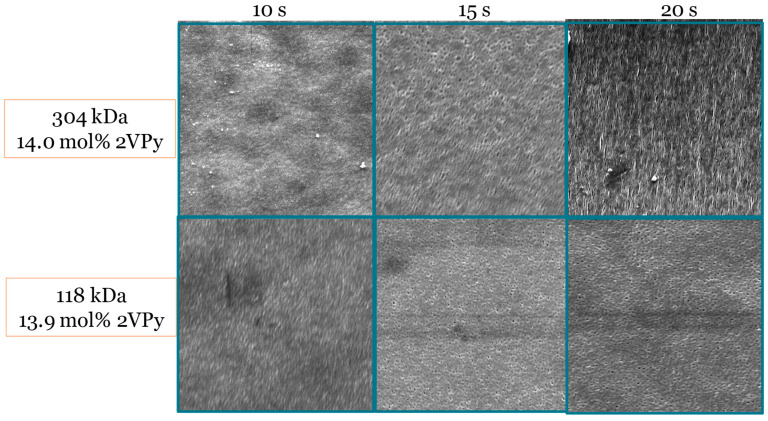
SEM images of membranes fabricated from 2VPy–Sty diblock copolymers at different evaporation times between film casting and immersion in the precipitation bath, while keeping the polymer concentration and solvent composition constant. All SEM images were acquired at a magnification of ×50,000. The images demonstrate the effect of evaporation time on membrane surface morphology and pore development. Representative pore size values determined from SEM image analysis are discussed in the text and summarized in Table 4.

The last parameter that was tested was the evaporation time before the immersion of the film into the water precipitation bath. As the evaporation time increased from 10 to 15 s, more ordered membrane structures with uniform pore size were observed with diblock copolymers. In the case of the polymer with the higher *M*_n_, a further increase in evaporation time led to the formation of irregular pores due to the lower mobility of this polymer compared to the diblock copolymer with the lower *M_n_*, for which no difference in membrane morphology was observed when the evaporation time was increased from 15 to 20 s. In general, when the evaporation time was equal to 15 s, a sphere percolation network was observed to form with both diblock copolymers. Figure 5 shows the SEM images of the fabricated membranes when the polymer concentration and solvent composition were kept constant, while the evaporation time before immersion in the water precipitation bath was varied between 10 and 20 s.

From the above optimization studies, 2VPy-*b*-Sty diblock copolymers with MWs ranging from 120 to 300 kDa and 2VPy contents between 10 and 15 mol% (and in optimal conditions) gave asymmetric polymeric membranes with a sphere percolation network morphology and pore diameters of around 50 nm. The membrane pore diameters determined directly using SEM and those calculated from water flux experiments are presented in Table 4.

***Membrane Performance.*** The produced membranes were characterized in terms of their water flux at a given applied pressure. The measured water flux was used, together with the Hagen–Poiseuille law for laminar flow through simple straight cylindrical pores, to obtain another estimation of the pore diameters. The Hagen–Poiseuille law states that

$$\dot{V}=\frac{\pi r^{4}∆P}{8nL}$$

where $\dot{V}$ is the volumetric water flux, *r* is the radius of the pore, Δ*P* is the pressure drop across the membrane, *η* is the viscosity of water, and *L* is the length of the pore in the top dense membrane layer. The determined volumetric water flux and the estimated pore diameter are also presented in Table 4. The table shows that the calculated pore diameters are very close to those directly determined using SEM, indicating that the pores were open and rather homogeneous. Although the membranes were prepared from diblock copolymers with similar PS/2VPy molar ratios, differences in molecular weight led to variations in pore diameter and pore surface density, which strongly affect water permeability. Given the r^4^ dependence of flux on pore radius, even relatively small differences in pore size result in large variations in volumetric water flux. Additionally, the volumetric water flux decreased with the decrease in the pore size, as expected.

**Figure 6:**
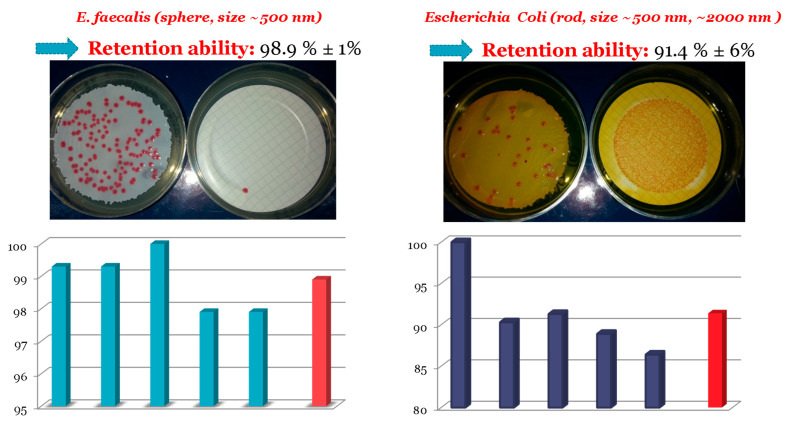
Histograms showing the repetition measurements of retention ability (blue bars) and the corresponding mean retention ability (red bar) for both microorganisms.

The performance of the membrane in retaining microorganisms that are harmful to human health was also tested by charging an aqueous suspension of microorganisms. Two such microorganisms with different shape were tested: *E. coli* and *Enterococcus faecalis*. *E. coli* is a common rod-shaped bacterium, while *E. faecalis* is spheroidal. After the filtration of the suspension, the polymeric membranes were placed in a selective medium and incubated at the proper temperature to develop pink colonies. The filtrate was filtered again using a commercially available 0.45 μm Millipore membrane that was also placed in a selective medium and incubated. The retention ability of the membranes was calculated from the number of colonies on the 2VPy-*b*-Sty-based membrane divided by the sum of the number of colonies on both the 2VPy-*b*-Sty membrane used for the original filtration and the Millipore membrane used for re-filtering the filtrate. Figure 6 presents the results of the repetition of retention ability for both microorganisms in blue, while the mean retention ability is presented in red.

Figure 6 shows that the retention ability for both microorganisms was higher than 90%, with the highest being that for *E. faecalis*. Furthermore, these high retentions concerned two microorganisms with various geometrical features. These results are very promising for the use of the present polymeric membranes for water treatment.

The toxicity of these polymeric membranes was also determined by spreading a certain load of migroorganisms onto the membranes. The determined toxicity calculated from the colonies placed on the membrane and the colony growth on the membranes was low and equal to 17%, proving that the membranes are not toxic. This suggests that, during filtration, no toxic substances migrate from the membrane to the water.

Recent studies have demonstrated that transport behavior and interfacial stability in functional membranes can be further tuned through synergistic ionic or molecular interactions in complex multicomponent systems. While the present work focuses on single-component diblock copolymer membranes, similar structure–property principles are evident. In our system, variations in molecular weight and block composition directly regulate the resulting network morphology, leading to distinct pore sizes, pore densities, and permeability values. These results highlight that even in relatively simple diblock copolymer systems, precise control over molecular architecture can effectively modulate transport properties, providing a clear platform for future extension toward more complex multicomponent or ionically interactive membrane systems, as recently reported in the literature [28].

## 4. Conclusions

High-molar-mass linear amphiphilic 2VPy-Sty diblock copolymers of various compositions and molar masses were successfully synthesized via controlled radical RAFT polymerization. Characterization of the linear diblock copolymers via AFM and DLS indicated the formation of spherical micelles with diameters between 188 and 378 nm in toluene. Subsequently, these diblock copolymers were successfully used for the preparation of well-ordered polymeric membranes via the phase inversion method. At optimal conditions, 2VPy-*b*-Sty diblock copolymers with molecular weight ranging from 120 to 300 kDa and 2VPy content between 10 and 15 mol% gave asymmetric polymeric membranes with a sphere percolation network morphology and pore diameters of around 50 nm. Evaluation of the fabricated membranes revealed a high retention of *E. faecalis* and *E. coli* and low membrane toxicity, thereby indicating that the developed membranes have the necessary attributes for water treatment.

## Figures and Tables

**Table 1:** Monomer conversion, molecular weight and composition characteristics of the synthesized 2VPy-styamphiphilic diblock copolymers. Rows highlighted in gray correspond to the homopolymer precursors.

Polymer Structure	2VPy (%mol)		Monomer Conversion (%)	MW_theor._^b^	GPC Results		
	Theory	^1^H-NMR			M_p_	M_n_	Ð
2VPy_222_^a^	100	100	74.7	23,500	44,500	38,200	1.09
2VPy_222_-*b*-Sty_1692_	10.1	11.6	72.9	199,000	248,000	154,000	1.54
2VPy_222_-*b*-Sty_1092_	19.6	16.9	76.0	137,000	205,000	108,000	1.57
2VPy_222_-*b*-Sty_315_	33.3	41.3	64.2	56,100	89,000	52,100	1.55
2VPy_222_-*b*-Sty_153_	51.7	59.2	59.2	39,200	54,100	40,500	1.50
2VPy_149_	100	100	49.5	15,600	47,500	32,400	1.33
2VPy_149_-*b*-Sty_915_	8.8	14.0	57.4	111,000	532,000	304,000	1.47
2VPy_234_	100	100	78.0	24,600	39,600	28,000	1.26
2VPy_234_-*b*-Sty_1449_	17.8	13.9	40	175,000	174,000	119,000	1.28

^a^ The subscript after the monomer represents the molar ratio of monomer to CTA in the final homopolymer, calculated from the initial monomer-to-CTA (feed) molar ratio (300 for 2VPy homopolymers) and measured monomer conversion. ^b^ Calculated from the DPs given in the polymer structure column (the CTA fraction attached to the polymer chain was taken into account).

**Table 2:** Hydrodynamic diameters of the micelles formed by the neutral and quaternized diblock copolymers in toluene and chloroform as measured using DLS and comparison with theoretical limiting values. Values shown in bold indicate the hydrodynamic diameters corresponding to the highest scattering intensity in each measurement.

Polymer Structure	D_hmax._ Theory	Chloroform	Chloroform + MeI	Toluene	Toluene + MeI
		D_h_ (nm)	D_h_ (nm)	D_h_ (nm)	D_h_ (nm)
2VPy_222_-*b*-Sty_1692_	964.7	**13.2**, 484.6	**14.3**, 65.5	**19.6**	18.1, **177.8**
2VPy_222_-*b*-Sty_1092_	662.3	**12.7**, 55.4	**13.0**, 74.6	18.1, **376.3**	**160.5**, 923.8
2VPy_222_-*b*-Sty_315_	270.6	**11.9**, 80.0	**11.4**, 67.1	**315.4**	33.2, **155.5**
2VPy_222_-*b*-Sty_153_	189.0	**10.1**, 41.2	**11.7**, 135.8	**188.7**	39.1, **146.3**

**Table 3:** Adsorption diameters of the micelles formed by the amphiphilic copolymers.

Polymer Structure	Micelle Diameter (nm)		
	Theoretical Maximum	Theoretical Minimum ^a^	AFM
2VPy_222_-*b*-Sty_1692_	964.7	29.8	348.1
2VPy_222_-*b*-Sty_1092_	662.3	24.7	328.3
2VPy_222_-*b*-Sty_315_	270.6	15.8	285.1

^a^ Determined utilizing randomized flight statistics adjusted for the carbon tetrahydral angle and employing PSty’s stiffness factor of 2.44 [28]. The formula applied to compute the root mean square diameter of gyration is expressed as follows: <dg^2^>^1/2^ = 2 × (2.44 × 2 × DP/3)^1/2^ × 0.154. Here, DP represents the degree of polymerization, and 0.154 nm signifies the length of a single carbon–carbon bond.

**Table 4:** Average pore diameter, average pore number surface density, experimental volumetric water flux, and theoretical pore diameters of the fabricated membranes.

Polymer Structure	SEM Results		Estimated Average Pore Length (nm)	Experimental Water Flux (L/h m^2^)	Calculated Pore Size (nm)
	Average Pore Diameter (nm)	Average Pore Number per m^2^			
2VPy_149_-*b*-Sty_915_	49.6	5.1 × 10^13^	190	31400	59.8
2VPy_234_-*b*-Sty_1449_	37.0	1.06 × 10^14^	190	10.8	38.4

## Data Availability

The original contributions presented in this study are included in the article. Further inquiries can be directed to the corresponding author.
